# Acceptability and Usability of a Reward-Based Mobile App for Opioid Treatment Settings: Mixed Methods Pilot Study

**DOI:** 10.2196/37474

**Published:** 2022-10-05

**Authors:** Steven L Proctor, Khary K Rigg, Allen Y Tien

**Affiliations:** 1 PRO Health Group Miami Beach, FL United States; 2 Thriving Mind South Florida Miami, FL United States; 3 Department of Psychiatry and Behavioral Health Herbert Wertheim College of Medicine Florida International University Miami, FL United States; 4 Department of Mental Health Law and Policy University of South Florida Tampa, FL United States; 5 Medical Decision Logic, Inc Baltimore, MD United States

**Keywords:** opioids, contingency management, mHealth, digital health, mobile app, innovation, opioid use disorder, recovery, acceptability

## Abstract

**Background:**

Contingency management is an evidence-based yet underutilized approach for opioid use disorder (OUD). Reasons for limited adoption in real-world practice include ethical, moral, and philosophical concerns regarding use of monetary incentives, and lack of technological innovation. In light of surging opioid overdose deaths, there is a need for development of technology-enabled solutions leveraging the power of contingency management in a way that is viewed by both patients and providers as acceptable and feasible.

**Objective:**

This mixed methods pilot study sought to determine the perceived acceptability and usability of PROCare Recovery, a reward-based, technology-enabled recovery monitoring smartphone app designed to automate contingency management by immediately delivering micropayments to patients for achieving recovery goals via smart debit card with blocking capabilities.

**Methods:**

Participants included patients receiving buprenorphine for OUD (n=10) and licensed prescribers (n=5). Qualitative interviews were conducted by 2 PhD-level researchers via video conferencing to explore a priori hypotheses. Thematic analysis of interviews was conducted and synthesized into major themes.

**Results:**

Participants were overwhelmingly in favor of microrewards (eg, US $1) to incentivize treatment participation (up to US $150 monthly). Participants reported high acceptability of the planned debit card spending restrictions (blocking cash withdrawals and purchases at bars or liquor stores, casinos or online gambling). Quantitative data revealed a high level of perceived usability of the PROCare Recovery app.

**Conclusions:**

Patients and providers alike appear receptive to microfinancial incentives in standard OUD treatment practices. Further pilot testing of PROCare is underway to determine acceptability, feasibility, and preliminary effectiveness in a rigorous randomized controlled trial.

## Introduction

America’s escalating opioid overdose crisis requires innovative solutions. Over 100,000 people died of drug overdose in 2021 in the United States—the majority of which involved opioids [1]. Contingency management (ie, rewarding people, often with monetary incentives, for achieving recovery goals) is an effective, evidence-based intervention for opioid use disorder (OUD) backed by decades of research [2] but remains highly underutilized. Motivational incentives are rarely used in real-world clinical practice due to several concerns, including most notably, the apparent lack of innovation, as well as moral, philosophical, ethical, and economic concerns, and even federal rules meant to prevent illegal inducements in health care [3]. Traditional contingency management protocols have become rudimentary, outdated, and onerous in the current digital era (eg, requirement for in-person appointments, use of a “prize bowl” filled with slips of paper), necessitating novel, technology-enabled solutions to facilitate widespread adoption. Many accepted contingency management procedures reward drug-free urinalysis screens exclusively, and there is only a low chance that the desired behavior will actually be rewarded in the commonly used probabilistic “prize-based” procedure in which patients earn draws from a prize bowl containing slips of paper with either no monetary value or a low-value prize when the target behavior is exhibited. This raises the common complaint that contingency management is a “game of chance” due to the lack of immediate and consistent meaningful reinforcement that is required for lasting behavior change.

Treatment programs may understandably voice concerns about increased costs associated with providing monetary rewards given that contingency management is often an “add-on” to usual care (ie, adjunctive intervention). However, research shows that contingency management, when combined with medication treatment for OUD, demonstrated the largest cost-savings relative to other evidence-based interventions for OUD, including medication alone [4]. A recent study examining the net impact of a digital therapeutic delivering contingency management via mobile app on medical costs due to hospital-based encounters and procedures among a sample of patients treated with buprenorphine for OUD documented that the medical cost reduction in patients using the app relative to those receiving standard care offset the cost of the digital therapeutic itself, thereby resulting in a net cost-savings of US $720 per patient [5].

Although there have been a number of recent strides in coverage for contingency management, including pilot programs in several US states, many commercial and government insurers remain slow to cover contingency management. There may also be legal concerns about whether the use of monetary incentives violates federal and state law because it could be considered unlawful to give money to patients who are enrolled in federally or state-funded health plans or programs. However, recent guidance from the Office of Inspector General (OIG) in the form of an advisory legal opinion (OIG Advisory Opinion No. 22-04) in March 2022 approved the use of a digital contingency management program using smartphone and smart debit card technology, which could clear the way for wider use of similar programs in routine treatment settings. The OIG has also dispelled the oft-stated assumption that the OIG bans contingency management incentives with a monetary value greater than US $75 [6].

Accumulating evidence suggests smartphone ownership, although certainly not universal, is no longer the barrier it once was [7-9]. In light of the increasing penetration of smartphone users, and the fact that many patients already leverage technology in all facets of their lives, reward-based apps have the potential to bring contingency management into the hands of more people receiving treatment for OUD. As a number of mobile apps begin to emerge in the treatment of OUD [10], there remain concerns about their quality, safety, potential efficacy, and availability of empirical evidence supporting their use for populations with OUD. Findings from a recent review study conducted to characterize currently available smartphone apps for the prevention, management, and treatment of opioid use, misuse, and related harm found that few apps meet basic quality standards [11], and even fewer reward-based apps have published peer-reviewed evidence regarding patient and provider perspectives on acceptability to inform uptake in real-world treatment settings. All currently available opioid-related apps were identified via web scraping of data from the Google Play and Apple App Store using the following keywords: *opioid use disorder, opioid abuse opioid misuse, opioid addiction, prescription opioid misuse, prescription opioid abuse, opioid abuse treatment, opioid abuse intervention, opioid abuse therapy, opioid abuse management,* and *opioid addiction recovery*. Of the 619 apps identified by the researchers, 59 apps met basic criteria for quality assessment, and only 1 app satisfied all standards on the screener for quality, as assessed by the American Psychiatric Association’s App Evaluation Model [12], which addresses the most fundamental questions to ask when considering using a digital health app (eg, *Does the app have a clinical/recovery foundation relevant to your intended use? Is there evidence of specific benefit from academic institutions, end user feedback, or research studies?*). Further work is warranted to fill this gap in technological solutions for OUD recovery management.

Guided by the Innovation Corps methodology, which uses the Lean Launchpad approach to developing hypotheses, then rapidly moving to continuous customer discovery with the aim of translating hypotheses into facts [13], this study evaluated PROCare Recovery, a multiplatform (iOS and Android), reward-based recovery management mobile app for patients receiving medication treatment for OUD. PROCare uses the power of motivational incentives and self-monitoring to reward people for achieving their recovery goals and engaging in their treatment plan. Development of PROCare was supported by a Small Business Innovation Research (SBIR) grant from the National Institute on Drug Abuse (NIDA). With an evidence-based reward system, people recovering from OUD can earn rewards for taking their medication as directed, attending appointments, taking routine self-report surveys to track recovery progress, completing science-backed learning modules in the psychoeducational library, as well as engaging in other recovery-oriented activities within the app. PROCare is a recovery management tool for people receiving medication treatment for OUD who are currently enrolled in outpatient treatment under the supervision of a clinician, and allows for many aspects of contingency management to be fully or partially automated, thereby addressing common logistical barriers to implementation. Patients have the opportunity to earn both monetary and nonmonetary rewards for completing various activities within the app as well as elements of their care plan such as Rapid Daily Check-In surveys (assessing craving, motivation, etc); more comprehensive monthly assessments (health care utilization, occupational functioning, quality of life, etc); taking their medication as directed; and accessing resources in the psychoeducational library to help educate, support, and encourage patients. Automated delivery of monetary rewards is achieved by depositing money to a pre-paid debit card with the option to apply spending restrictions. The “smart” debit card allows card administrators (eg, treatment program staff) to toggle specified blocking capabilities on/off to prevent cash withdrawals or purchases at identified high-risk vendors (eg, bars, liquor stores, casinos). Medication adherence and treatment engagement are translated to tangible financial rewards. With nonmonetary rewards, patients earn “credits” toward their “Degree(s) in Recovery” (associate, bachelor’s, master’s, and doctorate of recovery). Patients immediately earn monetary and non-monetary rewards for certain activities (eg, daily check-ins), whereas other activities first require verification before rewards are released. Medication adherence is confirmed by way of a combination of self-report and verification via urinalysis, and attendance at individual and group therapy appointments is confirmed via GPS location verification.

In light of surging opioid overdose deaths, there is a need for development of technology-enabled solutions leveraging the power of contingency management in a way that is viewed by both patients and providers as acceptable and feasible. This mixed methods study sought to determine the perceived acceptability and usability of PROCare Recovery, a reward-based, recovery monitoring smartphone app designed to automate contingency management by immediately delivering micropayments (eg, US $1) to patients for achieving recovery goals via smart debit card with blocking capabilities.

## Methods

### Recruitment

The current study was conducted as part of a phase I SBIR grant from the NIDA to build and test a reward-based recovery management smartphone app (PROCare Recovery) for patients with OUD receiving medication treatment. Participants were recruited from an addiction treatment system in South Florida. The study sample included licensed providers actively prescribing buprenorphine (n=5), and patients currently receiving buprenorphine for OUD (n=10). Participants had an average age of 41.93 years, and were predominately White, male, and employed full-time (Table 1).

**Table 1:** Sample demographic characteristics (N=15).

Variable		Value, n (%)
**Sex**		
	Male	9 (60)
	Female	6 (40)
**Ethnicity**		
	Hispanic	3 (20)
	Non-Hispanic	12 (80)
**Race**		
	White	13 (86.7)
	Asian	1 (6.7)
	Other	1 (6.7)
**Marital status**		
	Single	8 (53.3)
	Married	3 (20)
	Divorced	2 (13.3)
	Widowed	1(6.7)
	Separated	1 (6.7)
**Employment status**		
	Full-time	11 (73.3)
	Part-time	2 (13.3)
	Unemployed	2 (13.3)

### Measures

Individual in-depth qualitative interviews were guided by the Innovation Corps methodology given its emphasis on the Lean Launchpad approach to developing hypotheses, then rapidly moving to continuous customer discovery with the aim of translating hypotheses into facts [13]. A Project Advisory Board of addiction treatment industry experts, researchers, clinicians, administrators, people in recovery from opioid addiction, and family members of people with OUD, varying in background and expertise, was formed to inform the scientific and strategic direction of the phase I SBIR project. Following Project Advisory Board input with developing a list of hypotheses about the problem under study—here specifically regarding planned features and components of PROCare Recovery—semistructured interviews were conducted by two PhD-level researchers (authors SLP, KKR). In-depth interviews were selected for their ability to capture individual experiences and elicit detailed, granular responses. Participants were also administered the 10-item System Usability Scale (SUS) [14] as a quantitative measure of perceived PROCare usability (scores can range from 0 to 100 with >68 considered above-average). The SUS is a valid and reliable measure commonly used for global assessments of systems usability to evaluate a wide variety of products (mobile apps, software, websites, etc), including studies evaluating patient- and provider-facing mobile apps in addiction treatment settings [15-19]. The SUS can be accessed online [20].

### Procedure

After informed consent was obtained, participants were administered the semistructured interview, including a series of open-ended questions exploring a priori hypotheses, but space was made for the emergence of unanticipated themes allowing participants to fully describe their perceptions. Participants were asked for their thoughts on the acceptability of contingency management for routine use in addiction treatment settings in general, and perceived usability of the PROCare Recovery app in particular. Participants were informed that PROCare was a reward-based, technology-enabled recovery monitoring smartphone app delivering micropayments via smart debit card (True Link), with blocking capabilities for achieving recovery goals. Participants were shown the app and presented prompts with actual interview questions on the screen covering various topics and app features (ie, contingency management, outcomes monitoring, daily check-in, smart debit card system, psychoeducational library, appointment scheduling, target behaviors for rewards, and progress charting). Examples of questions included: (1) *What expectations do you have about an app designed to monitor outcomes for patients?* (2) *Do you have any reservations about the PROCare app?* (3) *What do you think of the features of the app?*

During the rewards section of the interview, participants were first asked how open they were to the idea of giving people small amounts of money (ranging from US $1 to US $5) for achieving their treatment goals, before being asked how much money in particular they thought it would take to properly incentivize participation each month. Participants were then asked a series of questions focused on the use of the True Link smart debit card system to facilitate delivery of monetary rewards. After participants provided their initial impressions regarding the smart debit card system, they were asked how important it was that such a debit card have blocking capabilities to prevent specified purchases. Participants offered their thoughts on what types of stores, businesses, or spending categories they believed should be automatically blocked for all card users in addiction treatment settings. Participants were also asked to comment on how they thought patients would feel about restrictions being placed on how they could spend the money earned from the PROCare app; that is, whether they believed such a strategy would be viewed as understandable or offensive to most patients receiving addiction treatment. Examples of questions included: (1) *What are your thoughts on the True Link smart debit card system?* (2) *How do you feel about restrictions being placed on how patients can use the money?* (3) *How receptive do you think patients will be to the non-monetary rewards?* At the conclusion of the interview, participants were administered the SUS to quantify perceived usability of PROCare. Interviews lasted approximately 60 minutes and were conducted via secure video platform (Zoom). Participants received a US $50 gift card for their participation. Interviews were recorded and automatically transcribed by Zoom. Transcripts were coded and thematically analyzed using Quirkos software by a PhD-level researcher with specialized training in mixed methods [21].

### Ethical Considerations

All study procedures were approved by the Medical Decision Logic, Inc Institutional Review Board (IRB00001558).

## Results

In general, our qualitative analysis revealed participants were very excited about the idea of an app that can help them in their recovery. Qualitative responses were consistent with SUS scores showing an exceptionally high level of perceived usability, as evidenced by a mean participant usability score of 92.2 (range 72.5-100). It was clear that participants perceived a need for a mobile app-based program and that PROCare Recovery seemed to be filling this void. Using such an app was only viewed as beneficial, and no major concerns about using the app were voiced.

When participants were asked for their thoughts about contingency management as delivered by PROCare, patients and providers were overwhelmingly supportive. There were no objections to giving patients small amounts of money for reaching their recovery goals. In fact, the idea of paying patients was generally described as “innovative” and “smart,” with very little concern being voiced. With regard to apparent benefits of using monetary rewards, one patient stated:

A lot of addicts will use the app just for the money, but it might save their life in the process.

The micropayment model in particular was described as “wise” because it did not give the patient “all the money at once.” The planned maximum monthly reward limit of US $150 was viewed as a reasonable amount for patients to earn monthly, as one patient explained:

People in recovery are broke. [US $150/month] is just the right amount. Not too much to hurt themselves. Small, which is good for cigarettes, food, and other things.

Further analysis revealed that participants believed the True Link debit card system was an appropriate safeguard. This was viewed as a superior option to giving patients actual cash, with some even describing the debit card system as a "necessity". The blocking capabilities were seen as a crucial piece to the debit card system and several suggestions were made regarding which stores, businesses, and activities should be blocked (eg, cash withdrawals, bars, liquor stores, casinos or online gambling, strip clubs).

## Discussion

### Principal Findings

This study provides important qualitative and quantitative evidence supporting the use of technology-enabled contingency management in real-world opioid treatment settings using a mobile app delivering rewards via smart debit card. Responses from in-depth interviews revealed that not only are patients themselves receptive to the idea of rewarding patients for achieving treatment goals, but providers also reported seeing value in the use of monetary incentives when proper safeguards are in place (blocking capabilities preventing ATM cash withdrawals, etc). Patients and providers both expressed enthusiasm for the micropayment model in which patients can earn small amounts of money (typically around US $1) for actively participating in their treatment (attending individual therapy appointments, producing negative urinalysis drug screens, attending community-based mutual-help support groups such as Narcotics Anonymous, etc), with the opportunity to earn a maximum monthly amount of US $150. Of particular interest, patients were in favor of the blocking capabilities, reporting that such spending restrictions “made perfect sense,” especially early in the recovery process. In addition to the qualitative findings, the observed SUS score of 92.2 far exceeds the industry standard of 68 indicating a high level of perceived usability of the PROCare Recovery app.

### Comparison With Prior Work

There has been rapid progress on the innovation front in recent years with respect to emerging mobile app technologies leveraging contingency management in the treatment of OUD [22-24]. The current findings contribute to the extant literature on promising reward-based mobile apps for OUD treatment populations and extend prior work by providing empirical evidence on patient and provider perspectives on the acceptability of providing motivational incentives in routine clinical practice. Several studies [25-27] have identified concerns shared by patients as well as providers tasked with implementing contingency management, including the overreliance on abstinence, fairness, perceived power imbalance, and how incentives will be spent. One of the most commonly identified concerns about contingency management is how patients will use the money earned (ie, “giving people ‘extra’ money at a vulnerable point in their treatment pathway may do more harm than good”) [27,28]. The current study findings, however, demonstrate that contingency management protocols using “micro” rewards and a mobile app with accompanying “smart” debit card technology—where blocking capabilities and spending restrictions can be put in place—is viewed by both patients and providers as an appropriate safeguard and a critical piece to any reward system.

### Limitations

This study has several limitations, including most notably, a relatively small sample size, thereby limiting the generalizability of the findings. Although a small sample is generally acceptable for a focused, qualitative pilot study designed to inform preliminary technology development, conclusions drawn from such a small sample require replication in a larger scale study. The study sample was also predominately White and did not include any Black, American Indian, or Pacific Islander participants. Further research with a larger, more racially diverse population is warranted. Finally, although this study was able to collect useful data on perceived feasibility of PROCare by way of in-depth qualitative interviews with both patients and providers, a logical next step for future work is to assess for additional indicators of feasibility, including patient access to smartphone technology, level of digital health literacy, and comfort with technology.

### Conclusions

Notwithstanding sample size limitations, current findings suggest strong preliminary evidence that both patients and providers alike appear to be in favor of contingency management for OUD, particularly when using a micropayment model delivered via mobile app with smart debit card and spending parameters for monetary rewards earned. Although patients and providers reported a high level of perceived usability of the PROCare Recovery app, further testing in a large-scale randomized controlled trial is necessary to determine preliminary effectiveness of the PROCare Recovery app as a solution to enhance medication and care plan adherence, and ultimately improve outcomes for OUD treatment populations.

## Abbreviations

NIDA: National Institute on Drug Abuse
OIG: Office of Inspector General
OUD: opioid use disorder
SBIR: Small Business Innovation Research
SUS: System Usability Scale
